# An efficient estimation of crop performance in sheep fescue (*Festuca ovina* L.) using artificial neural network and regression models

**DOI:** 10.1038/s41598-022-25110-8

**Published:** 2022-11-28

**Authors:** Masoomeh Abbasi Khalaki, Esfandiar Jahantab, Moslem Abdipour, Mehdi Moameri, Ardavan Ghorbani

**Affiliations:** 1 Department of Range and Watershed Management, University of Mohaghegh Ardabili, Ardabil, Iran; 2 Department of Range and Watershed Management, Faculty of Agriculture, Fasa University, Fasa, Iran; 3 Kohgiluyeh and Boyerahmad Agricultural and Natural Resources Research and Education Center, Agricultural Research, Education and Extension Organization (AREEO), Yasuj, Iran; 4 Department of Plant and Medicinal Plants, University of Mohaghegh Ardabili, Ardabil, Iran

**Keywords:** Plant sciences, Biological techniques

## Abstract

*Festuca ovina* L. (sheep fescue), a perennial grass plant found in mountainous regions, is important from both an ecological and economic viewpoint. However, the variability of biological yield of sheep fescue due to its reliance on different characteristics makes it difficult to accurately prediction using classic modeling techniques. In this study, machine learning methods and multiple regression models (linear and non-linear) are used to investigate the interdependence of various morphological and physiological characteristics on accurate prediction of the biological yield (BY) of sheep fescue. Principal components analysis and stepwise regression were used to select six agronomic parameters i.e. thousand seed weight (TSW), relative water content (RWC), canopy cover (CC), leaf area index, number of florescence, and viability (VA), while the output variable was BY. To optimized the artificial neural network (ANN) structure, different transfer functions and training algorithms, different number of neurons in each layer, different number of hidden layers and training iteration were tested. The accuracy of the models and algorithms is analyzed by root mean square error (RMSE), mean absolute error (MAE), and determination coefficient (R^2^). According to the findings, ANN models were more accurate than regression models. The ANN model with two hidden layers (i.e. structure of 6–4–8–1) which had RMSE, MAE and R^2^ scores of 0.087, 0.065 and 0.96, respectively, was discovered as the best model for predicting the BY. In addition, result of the sensitivity analysis showed TSW, RWC and CC, in that order, were the variables most important for high-quality BY estimation in both models regardless of input combination. Finally, the paper concludes that early flowering sheep fescue genotypes with long maturation and great TSW must be regarded as the most suitable model for increasing BY in breeding projects.

## Introduction

*Festuca ovina* L. (sheep fescue) is a perennial grass plant with good quality for grazing^1^. The main *F. ovina* usage is for cultivation as forage in rangelands revegetation programs^2^. The plant is a densely tufted and is a drought-resistant grass^3^. Additionally, *F. ovina* has a strong capacity to improve water and nutrient absorption in poor soils due to mycorrhizal fungi^4^. According to the above, one of the most important goals for pasture managers is to enhance sheep fescue yield using appropriate farming practices However, timely and cost-effective evaluation of plant performance characteristics is essential for the management and exploitation of this valuable plant. It is difficult to estimate biological yield since it is a polygenetic trait that is highly influenced by the environment and has a low heritability. One technique for addressing this subject is to predict and model this trait through other tarits with higher heritability that affect biological production either directly or indirectly in a positive or negative way^5^.

For the analysis and prediction of biological yield, a variety of methods have been offered; these methods can be chosen depending on the objectives, type, and complexity of correlations between traits^6^.

According to literatures review, there are no modeling studies in sheep fescue to predict biological yield. In relation to other plant species, modeling studies have been performed for some plant species, with an emphasis on techniques based on linear relationships between parameters, such as path analysis (PA), multiple linear regression (MLR) and correlation^7–10^. These approaches, however, are limited to linear correlations and are unable to capture nonlinear and complex interactions between variables. In the presence of nonlinear effects, it appears that these approaches to be incapable of providing a complete and accurate illustration of the interactions between biological yield and its elements^11,12^.

Artificial intelligence techniques such as artificial neural networks (ANNs), genetic expression (GE), Bayesian classification (BC), adaptive neuro-fuzzy inference system (ANFIS), and other advanced modeling methods, in opposed to previous modelling techniques such as PA and MLR, have lately gotten a lot of attention from crop researchers, particularly when the relationship among parameters may well be nonlinear^5,13–15^. Artificial Neural Network (ANN) is a multi-networked (multilayer perceptron) system of logically arranged fundamental units that simulate the neuron activity in the human brain. It may model the complex and non-linear relationships without any prior assumptions of cause and effect relationships of the variables and comparatively advanced and competitive as compared to conventional regression and statistical models^16^. Neural network techniques are being effectively applied in many domains including agriculture, engineering, medical sciences, etc. In agriculture, ANN has been applied in predicting the biological yield for grass pea^17^. They found that the ANN models with the same input variables could predict the biological yield with a higher performance (R^2^≈ > 0.92) compared to the MLR models (R^2^≈ < 0.65). This advantage can be due to the nonlinear or complex relationships between variables and the high ability of nonlinear functions to find and capture them in ANN models. Mokarram and Bijanzadeh^18^ also compared MLR and ANN including multi-layer perceptron (MLP) and radial basis function (RBF) models to predicting biological yield of barely. Among the MLR, MLP and RBF models, MLP model had the highest R^2^ values for.

prediction of BY (R^2^ = 0.894).

Although, many researches have been performed to specify the best models for predicting biological process/plants yield using different types of data^19,20^. Abdipour et al.^5^ expressed ANN forecasted safflower seed performance with greater precision and effectiveness than the multi linear regression method. According to Ghodsi et al.^21^, the ANN model is an effective tool for predicting wheat production. Moreover, Mutlu et al.^22^ reported that NIR combined with the ANN, were able to accurately predict the characteristics of wheat flour. Safa et al.^23^ stated that Model ANN accomplished better than Model MLR in predicting CO_2_ emissions.

Based on the literatures there are various reports about the capability of the different modeling methods, and it has been reported that ANN always executed better than MLR or other models^24^. So, modeling the performance of sheep fescue based on its factors would be beneficial to understand the relationships between the most important organs of the plant in order to improve cultivation and harvesting programs.

Although in some studies the relationships between traits in this plant have been investigated with methods such as correlation^25^, modeling for biological function in this plant has not been done either by classical methods such as regression or by artificial intelligence methods. Therefore, determining the relationships between traits and an optimal model for predicting biological yield using strong modeling methods such as ANNs can greatly help breeders to improve the biological yield of this plant.

To the best knowledge of the authors, no research has performed a robust analysis on the interdependence of morphological and physiological attributes and biological yield in sheep fescue, and predicting of BY through the corresponding most suitable ANN algorithms.

According to the above, the purpose of the present study was to investigate and predict sheep fescue performance based on its morphological and physiological characteristics using ANN, MLR and non-linear regression models.

## Materials and methods

### Study area and climatic characteristics

**Figure 1:**
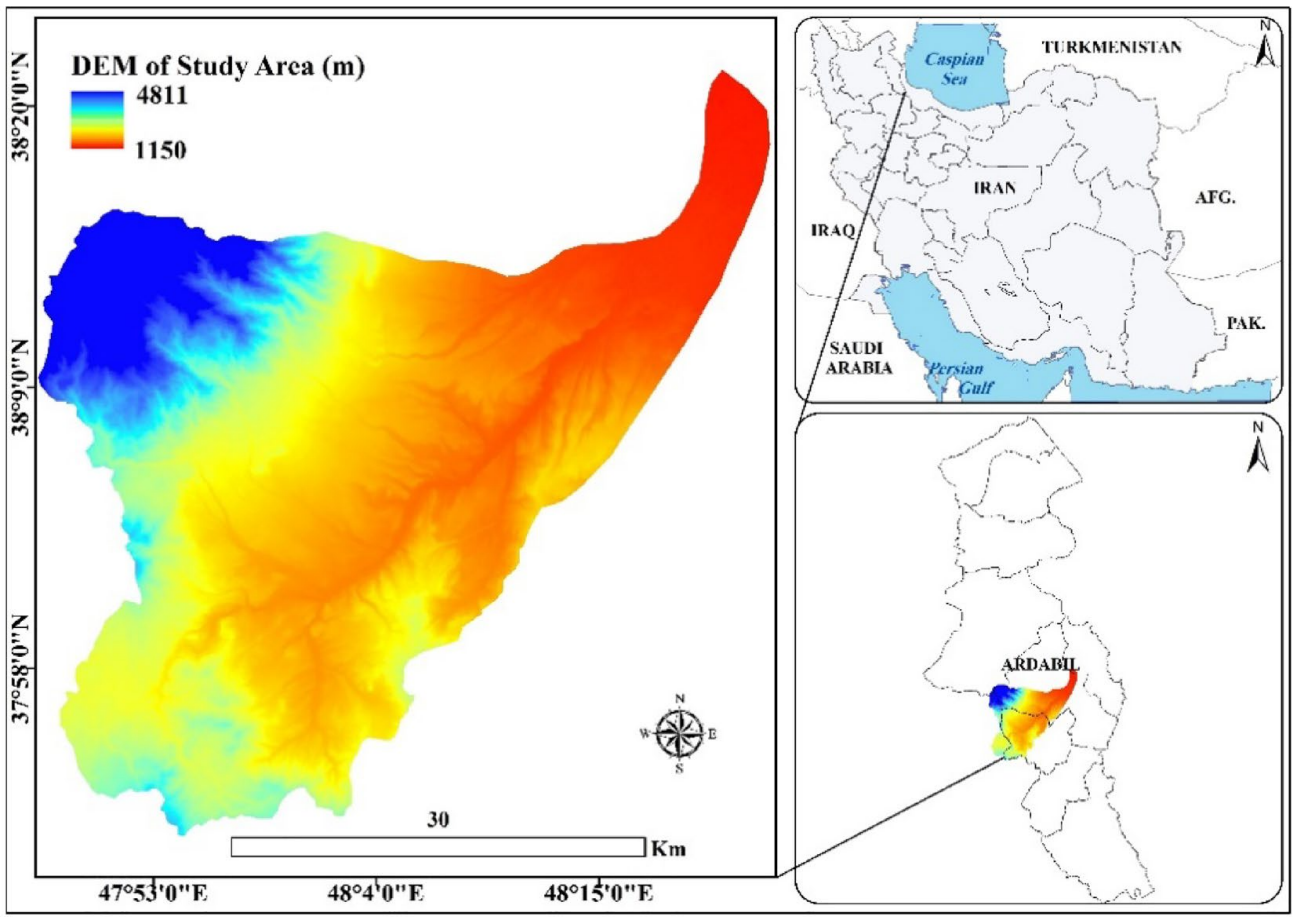
The location of the study area.

The present research was performed under rain-fed conditions at two planting times in spring and autumn 2018 in the field of Balekhlichay watershed dry-farming lands in an area about 1058 km^2^, in Ardabil province, northwest of Iran (38°12′44″ N and 48°17′46″ E with altitude range from 1150 to 4811 m above sea level) (Fig. 1). The mean annual rainfall and mean temperature range at the low and high altitude are 299–766 mm and 3.9–7.9 °C, respectively and the study area slope is at the range of 12–60%. The dry-farming lands used in this research were among the lands of University of Mohaghegh Ardabili, Iran and because this research was supported by the Vice-Chancellor for Research and Technology of the University, did not need to obtain a permission.

The soil is characterized by a pH of 6.7–8.8, total mobile nitrogen of 2–36% and the electrical conductivity (EC) of 0.18–0.24 dS/m, available potassium of 170–217 ppm, available phosphorus of 20.58–33.61 ppm, C/N ratio of < 15, organic carbon (OC) of 0.07–3.8%, clay content of 17%, sand content of 69%, and silt content of 14%.

### Field experiment, growing conditions and treatments

This study was done as a factorial experiment based on a randomized block design (RCBD) with three replications. The experimental factors were (1) planting time in two seasons (autumn and spring) and (2) facilitators in nine levels, including control, PSN (500 and 1000 mg/lit), EM (1 and 2%), Super absorbent (10 and 30 g/kg soil), Organic fertilizer (cattle manure) (100 and 200 g/kg soil). Table 1 shows the characteristics of the growth facilitators used in the present study. After determining the cultivation area, first, the ground plowed,the weeds were removed and the cultivation bed was prepared. Each plot was split into three subplots. Within each subplot, 15 holes were drilled, and 5 seeds were seeded. The seeds of the *Festuca ovina* L. were collected from its habitat in Sablan rangelands, Ardabil, Iran (47° 52′ E and 38° 12′ N). After harvesting from rangelands, seeds of *Festuca ovina* are stored in the herbarium of Faculty of Agriculture and Natural Resources, University fo Mohaghegh Ardabili with number 1342. It should be mentioned that harvesting the seeds of rangeland plants from the rangeland of Iran is free, however, the permission to harvest the seeds of the *Festuca ovina* from the Sabaland rangelands was obtained from the Department of Natural Resources and Watershed Management of Ardabil province, Iran. According to two planting seasons, 9 fertilizer combinations, dividing each plot into three plots and three replications, 162 data were collected, 18 outlier data were removed and 144 data were used for analysis.

**Table 1:** Some characteristics of PSNs, EM, Superabsorbent and Organic fertilizer.

**PSNs**	
Molar mass	84/9947 (g/mol)
Density	2/257 (g/c^3^ at 16 °C)
melting point	380 (°C)
Boiling point	380 (°C)
Specific heat capacity	95/06 (J/mol-K)
Particle size	120 (Nm)
Solubility in water	730 (g/l at 0 °C)
	921 (g/l at 25 °C)
	1800 (g/l at 100 °C)
Appearance	White powder or crystal
**EM**	
Type of biotic fertilizer	Effective microorganisms
Number of EM in the composition (C^3^)	120
pH	4 >
Compounds	Water + Sugarcane Molasses + Aloevera + Photosynthetic Bacteria + Lactic acid bacteria + Yeasts
**Superabsorbent**	
Name	Bolour Ab
Appearance	Brown powder
Moisture (%)	5 >
Particle size (μm)	200–400
pH	6–7
Water absorption capacity (ml/g)	500
mass density (g/cm^2^)	0/8
Effective longevity in soil	2–7 years
Water usable for the plant	> 95%
Solubility	Insoluble in water and organic solutions
Odor and toxicity	–
**Organic fertilizer**	
Type of manure	Cow manure
Total N (%)	0/57
P (%)	0.09
K (%)	1/1
EC (dS m^−1^)	6/1
pH	6/8

#### Applying of treatments and maintenance

**Figure 2:**
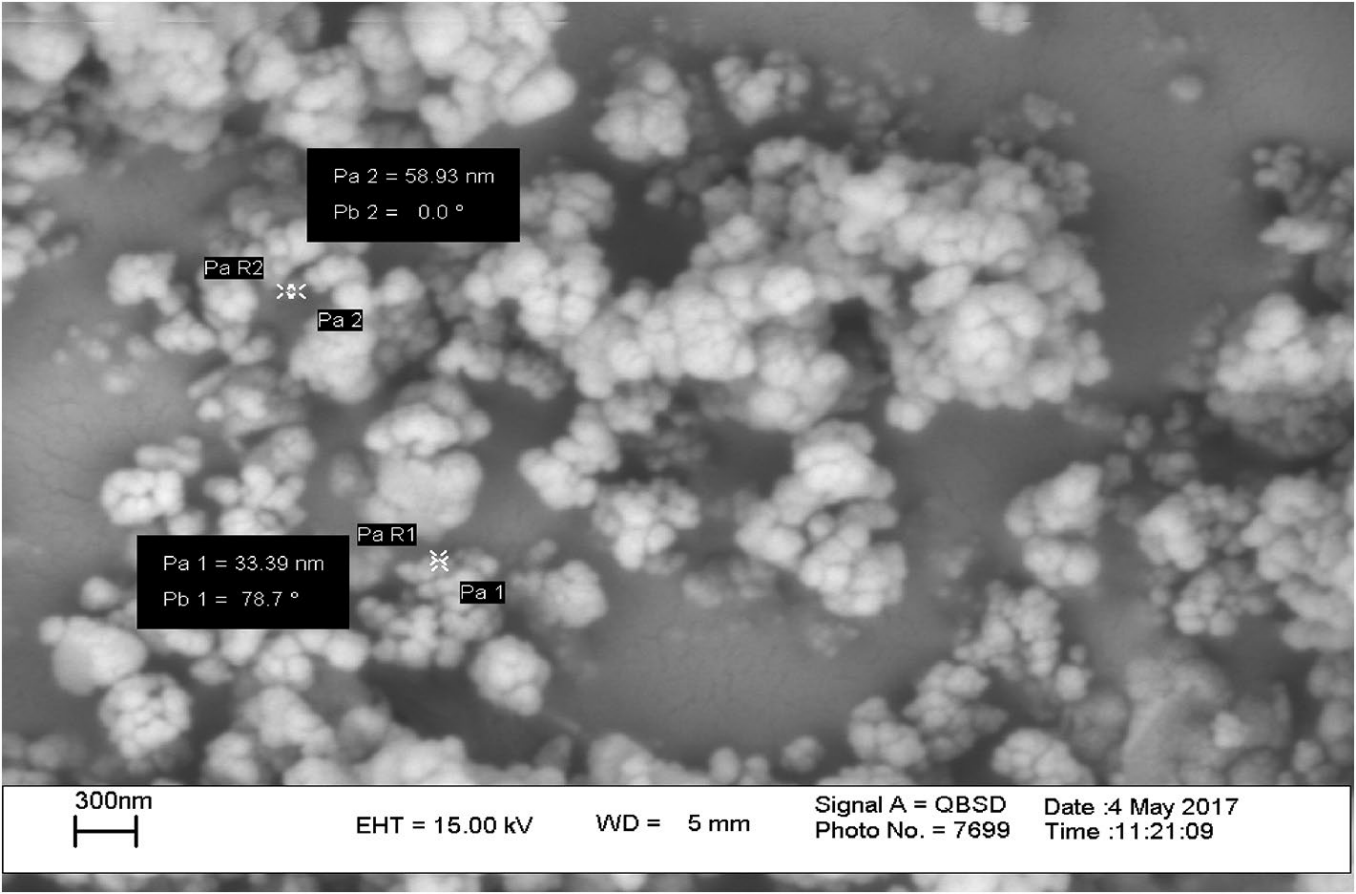
Scanning electron microscopy (SEM) image of potassium silicate nanoparticles.

Powder of potassium silicate nanoparticles (PSN), effective microorganisms (EM) and Super absorbent (SA) were supplied by Sigma Aldrich Company (Fig. 2), Emkanpazir Pars Company (Shiraz, Iran), and Bojnourd Water Crystal Production Company, respectively. The morphological study of these nanoparticles was conducted by scanning electron microscope (SEM). The superabsorbent polymer used in this research was purchased from North Khorasan Bolour Ab Company (Shirvan, Iran). Organic fertilizer (cattle manure) was prepared from the green space of University of Mohaghegh Ardabili.

When the plants had four primary leaves, PSN and EM, were added to the soil of each clump as solution. Treatments of PSN and EM in three steps and ten days apart were added to soil as solution. Super absorbent and Organic fertilizer were added to the soil. For the Super absorbent treatment, 10 and 30 g of Super absorbent separately were mixed with 1 kg soil and pits were filled with them. For the Organic fertilizer treatment, 100 and 200 g/kg of cow manure were combined with soil and were poured in the pits. During the growing season, weeds were removed mechanically, without the use of chemical pesticides.

### Field sampling and measurement

Three randomly chosen plants from each plot were evaluated for harvest plant information using indicators including plant height (PH) (At the end of the growth period, the length of the shoots and roots of the plant were harvested and cleaned, then measured with a precise ruler), basal diameter (BD) (The basal diameter of harvested plants was measured with a precise ruler), canopy cover (CC) (First, the geometric shape of the circle or oval of the canopy was recognized, then they were calculated based on the formulas of the area of the circle and oval) and etc. Ten morpho-physiological traits including plant height (PH (, basal diameter (BD), canopy cover (CC), number of florescence (NI) (At the end of the vegetative period, the number of inflorescences was measured), thousand seed weight (TSW) (The thousand seeds of each species was considered randomly and accurately obtained using a digital scale), viability (VA) (It was considered based on the number of holes in which seed cultivation was done. Total Chlorophyll (TCh) (The amount of chlorophyll in plant leaves was measured by a chlorophyll meter)^26^, leaf area index (LFA) (leaf area index was measured based on ground based method)^27^, relative water content (RWC)^28^ and biological yield (BY)^29^ were evaluated in this study. For this purpose, in the end of growth stage, 3 plants from each plot were randomly selected^30,31^. Summary of statistical indices for estimated characteristics are shown in Table 2. The relative water content of leaf was specified by the following equation.

$${\text{RWC}} = \left( {{\text{FW}} - {\text{DW/SW}} - {\text{DW}}} \right) \times {1}00$$

where FW: fresh leaf weight immediately after sampling, DW: dry weight of leaf after drying in oven and SW: saturated leaf weight after placing in distilled water.

**Table 2:** Summary of statistical indices of estimated characteristics.

Characteristic	Min.	Max.	Mean ± SD
PH (cm)	15	35	22.04 ± 4.54
BD (cm)	2	15	8.11 ± 2.93
CC (cm^2^)	1	6.75	4.35 ± 1.45
NI (N m^-2^)	1	8	4.26 ± 1.15
TSW (g m^-2^)	0.10	0.30	0.2 ± 0.04
VA (%)	8	33	22.08 ± 5.04
TCh (mg g^-1^)	1.6	18.22	7.06 ± 4.14
LAI (cm^2^)	2.12	11.15	7.04 ± 2.12
RWC (%)	1.64	10.55	6.54 ± 2.06
BY (g m^-2^)	10.11	63.11	40.74 ± 14.59

PH: Plant height, BD: Basal Diameter, CC: Canopy Cover; NI: Number of florescence; TSW: Thousand seed weight; VA: Viability; TCh: Total Chlorophyll, LAI: Leaf area index; RWC: Relative Water Content of Leaf; Biological yield (BY).

### Data preprocessing and statistical analysis

In order to avoid bias’s estimation due to differences in units of input variables, the following equation was used to normalization within in the ranges [0.1, 1] (Eq. 1).

1 $${\text{x}}_{{\text{n}}} = \left[ {\left( {\frac{{{\text{x}}_{{\text{i}}} - {\text{x}}_{\min } }}{{{\text{x}}_{\max } - {\text{x}}_{\min } }}} \right) \times 0.9} \right] + 0.1$$

where x_i_ is the original value, x_n_ is the normalized value, and x_max_ and x_min_ are the max and min values, respectively.

The nature and size of connections between BY and other qualities were investigated using a multiple linear regression (MLR) model and nonlinear regression models such as Exponential, Logistic, Quadratic, Gompertz, Asymptotic exponential, and Chapman-Richard. We chose the MLR model as the best regression model to characterize the correlations between variables based on model performance results (Table 5). As a result, the MLR model was used as a starting point for additional research. It should be noted that the most important hypotheses for selected MLR model including the existence of a linear relationship between the dependent and independent parameters, the independence of errors across the independent variables and homoscedasticity (Durbin-Watson value of 1.83), the absence of multicollinearity between the predictor factors (variance inflation factor (VIF value < 5) and tolerance (TOL value > 0.2) were evaluated (Table 6). SAS Institute Inc's statistical analysis system (SAS software) version 9.4 was used to assess the hypotheses and normalcy.

### Input parameters selection

Input variable selection (IVS) is a crucial phase in the creation of a statistical model that has a significant impact on the model's performance. In general, when a high set of input parameters are utilized for a small sample size, researchers typically use models with the fewest input variables to answer their problems^14,32^. The simple correlation as an input variable selection method only shows the magnitude of the relationship among attributes and does not provide clear information about different kinds of direct or indirect effects among them. This drawback is important because the correlation coefficient between two variables can be affected by indirect effects of other variable(s) in a positive or negative way, reducing the chance of achieving a unique solution^15^. Because correlation analysis is ineffective as an IVS method, we employed principal component analysis (PCA) and stepwise regression (SWR), two more well-known and powerful IVS approaches (Tables 3 and 4). Both SWR and PCA are methods to separate the variables influencing the dependent variable for modeling to reduce the data volume. In the PCA, a linear combination of independent variables with the highest relationship with the dependent variable is determined, and usually this linear combination justifies a high percentage of changes in the dependent variable. In the SWR method, the variables with the highest correlation with dependent variable are entered into the model, and in the final stage, a model with a combination of the most influential variables is obtained.

**Table 3:** Stepwise regression analysis for biological yield as the dependent variable of *F. ovina.*

Step	Entered variable	Variables in Model	Partial R-Square	Model R-Square	Model Adjusted R-Square
1	TSW	TSW	0.252	0.252	0.246
2	RWC	TSW, RWC	0.190	0.442	0.433
3	CC	TSW, RWC, CC	0.129	0.571	0.558
4	LAI	TSW, RWC, CC, LAI	0.101	0.672	0.649
5	NI	TSW, RWC, CC, LAI, NI	0.085	0.758	0.731
6	VA	TSW, RWC, CC, LAI, NI, VA	0.058	0.815	0.786

CC: Canopy cover; NI: Number of florescence; TSW: Thousand seed weight; VA: Viability; LAI: Leaf area index; RWC: Relative Water Content of Leaf.

**Table 4:** Principal component analysis in *F. ovina.*

Variable	PC1	PC2	PC3	PC4
PH	0.104	0.124	0.265	0.078
BD	0.085	0.036	− 0.400	0.343
CC	0.353	− 0.191	− 0.196	− 0.464
NI	0.339	− 0.027	− 0.409	0.271
TSW	0.364	− 0.063	− 0.090	− 0.453
VA	0.366	0.058	− 0.073	− 0.339
_TCh_	0.105	0.176	0.722	− 0.022
LAI	0.339	− 0.418	0.120	0.383
RWC	0.337	− 0.434	0.118	0.347
Eigenvalue	6.78	0.93	0.57	0.30
Justified variance (%)	0.75	0.10	0.06	0.03
Cumulative variance (%)	0.75	0.86	0.92	0.95

PH: Plant height, BD: Basal Diameter, CC: Canopy coverage; NI: Number of florescence; TSW: Thousand seed weight; VA: Viability; TCh: Total Chlorophyll, LAI: Leaf area index; RWC: Relative water content.

### Multiple linear regression

By proving the superiority of multiple linear regression over other regression models and the validity of multiple regression assumptions in the previous section, this regression model was calculated with the following equation (Eq. 2):

2 $$y_{i} = \beta_{0} + \beta_{{1}} x_{1} + \beta_{{2}} x_{2} + ... + \beta_{{\text{n}}} x_{n} + \varepsilon_{i}$$

where y_i_ is the biological yield, $\beta_{0} - \beta_{n}$ are the regression coefficients, ${\text{x}}_{{1}} {\text{ - x}}_{{\text{n}}}$ are input factors, and $\varepsilon$ is error associated with the observation.

### Artificial neural network

A “Multi-Layer Perceptron (MLP)” model was constructed and the ANN model was calibrated with the help of MATLAB, R2018a, to build and analyze the efficacy of ANN to forecast the biological yield of sheep fescue^33^. The following equation represents the output of the ANN model in this research^34^:

3 $$y_{{\text{t}}} = \alpha_{0} + \sum\limits_{{{\text{j}} = 1}}^{{\text{n}}} {\alpha_{{\text{j}}} {\text{f}}} \left( {\sum\limits_{{\text{i = 1}}}^{{\text{m}}} {\beta_{{{\text{ij}}}} y_{{\text{t - 1}}} } + \beta_{{{\text{0j}}}} } \right) + \varepsilon_{{\text{t}}}$$

where $y_{{\text{t}}}$ is the network output (BY), ${\text{n}}$ is the number of hidden nodes, ${\text{m}}$ is the number of input nodes, ${\text{f}}$ is tangent sigmoid transfer function, $\alpha_{j} \{ {\text{j = 0,1,}}...{\text{,n}}\}$ symbolize the weight vectors from the hidden to the output nodes, $\beta_{{{\text{ij}}}} \{ {\text{i}} = 1,2,...,{\text{m}};{\text{j = 0,1,}}...{\text{,n}}\}$ are the input weights to the hidden nodes, and $\alpha_{0}$ and $\beta_{{0{\text{j}}}}$ are the arc weights that lead from the bias terms, which are always equal to one.

**Figure 3:**
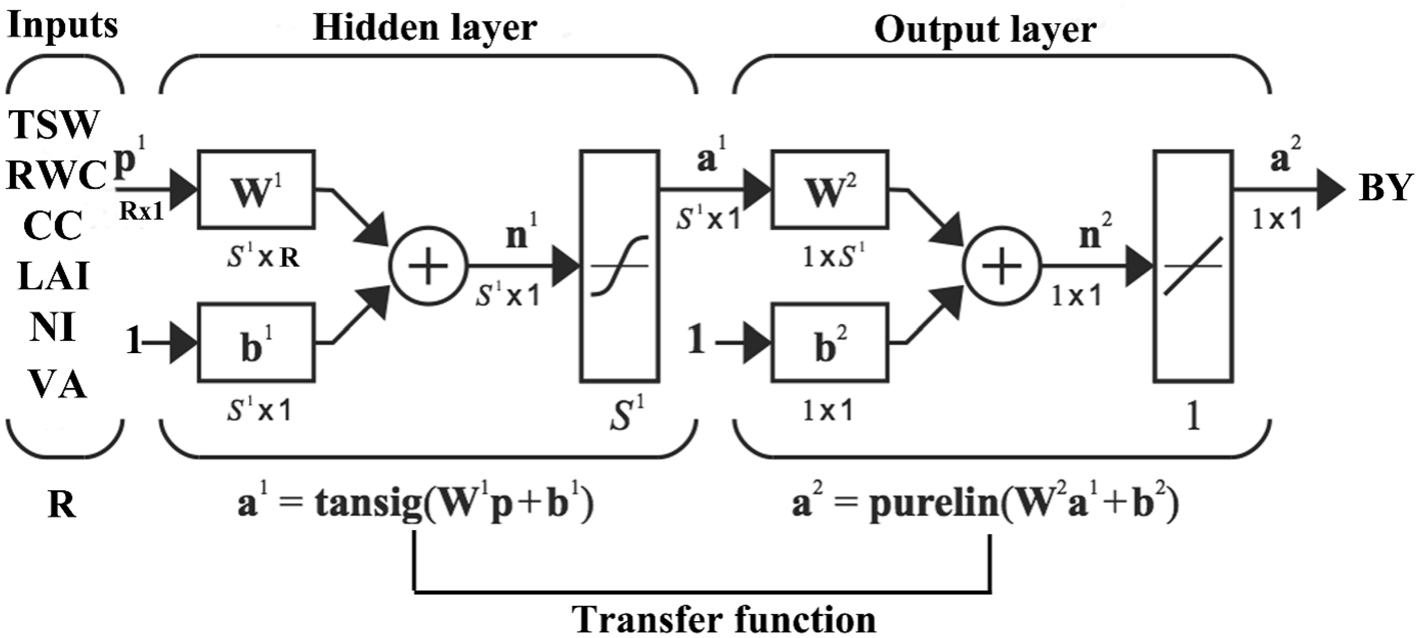
Applied structure of MLP model to predict BY.

The initial fine-tuning of ANN topology was optimized by changing the hidden layers (1–3 layer), neurons in each hidden layer (1–30 neurons/layer), learning algorithms (Levenberg–Marquardt, Momentum and Conjugate gradient), transfer functions for hidden layer (Tansig, logsig and purelin), and the most effective network structure was created (Fig. 3).

**Figure 4:**
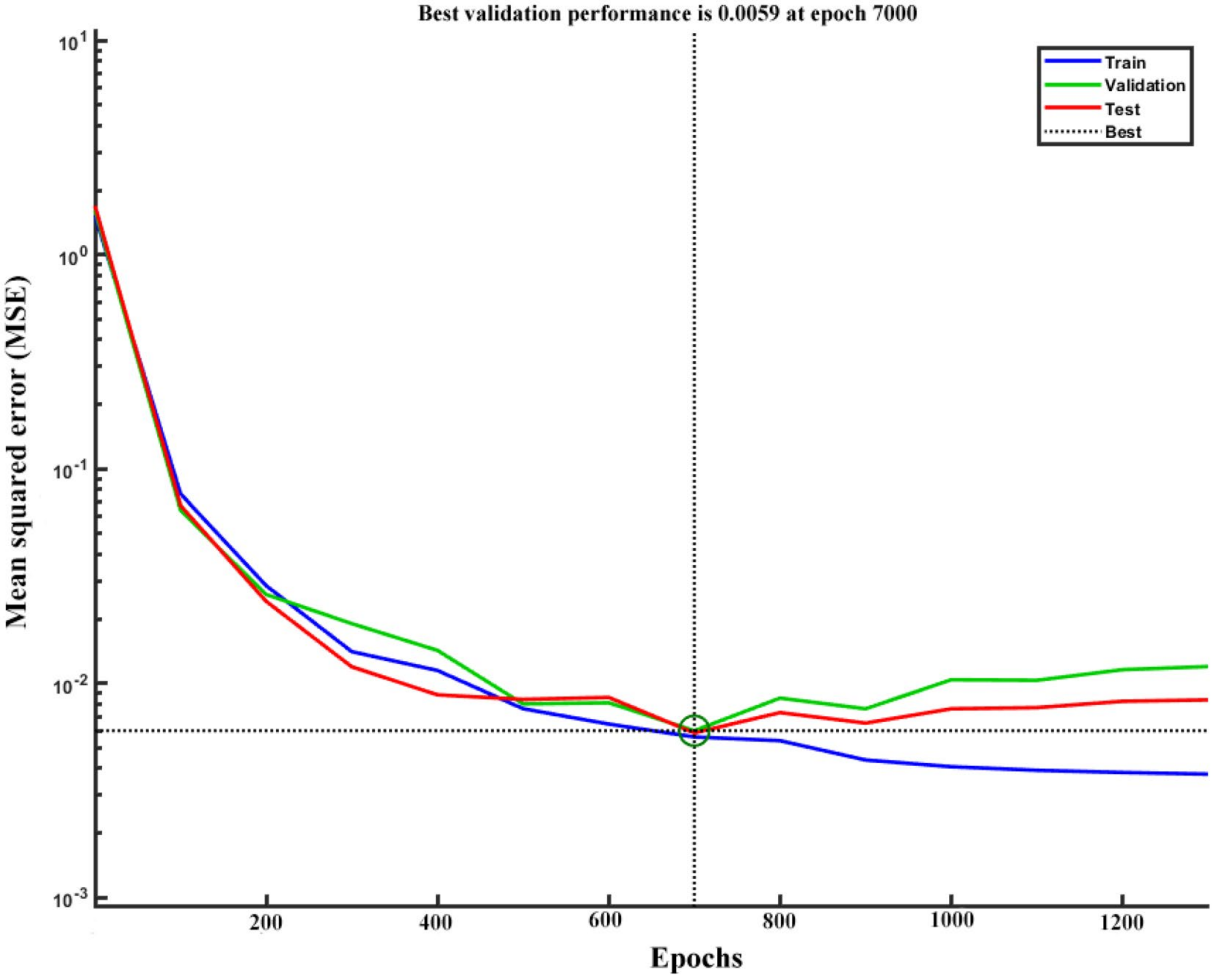
The convergence of the average of MSE value during training and cross validation of the final ANN structure.

Each run on the training dataset was performed with a 2000 epoch size (training cycles) and a mean square error (MSE) cutoff value of 0.01 (based on normalized dataset scale). The mean of MSE values during the training, testing, and cross-validation stages in different epochs (1–2000) was explored to obtain an appropriate training procedure and avoid overtraining. Around 700 epochs are sufficient for convergence between training, testing, and cross-validation, as illustrated in Fig. 4. The entire dataset in this study was 144, which was randomly divided into three subsets: training (65 percent), testing (20 percent), and validation (15 percent)^35^. Due to the MATLAB software generates various random data for each run, the best ANN for each topology was chosen after a maximum of 40 runs.

### Model implementation and sensitivity analysis of input parameters

Three statistical quality indicators, namely mean absolute error (MAE), root mean square error (RMSE), and coefficient of determination (R^2^), were utilized to objectively examine the efficacy of ANN and MLR models to predict the biological yield of sheep fescue according to its variables.

4 $${\text{RMSE = }}\sqrt {\frac{{\sum\nolimits_{i = 1}^{n} {(O_{i} - P_{i} )^{2} } }}{n}}$$

5 $${\text{MAE = }}\frac{1}{n}\sum\nolimits_{i = 1}^{n} {\left| {O_{i} - P_{i} } \right|}$$

6 $${\text{R}}^{2} = \frac{{\sum\nolimits_{i = 1}^{n} {(O_{i} - \overline{O})(P_{i} - \overline{P})} }}{{\sqrt {\sum\nolimits_{i = 1}^{n} {(O_{i} - \overline{O})^{2} \sum\nolimits_{i = 1}^{n} {(P_{i} - \overline{P})^{2} } } } }}$$

where *n* is the number of data, *O*_*i*_ denotes the observed values, *P*_*i*_ denotes the anticipated values, and the bar represents the variable's mean.

A sensitivity analysis was used to examine the impact of several independent factors on the outcome. Sensitivity analysis reveals the usefulness of each variable, and can be used to identify the components that are most important for forecasting output^36^. For this, the dataset was run without any input variables (i.e., TSW, RWC, CC, LAI, NI, VA), and the models' implementation was assessed using R^2^, RMSE, and MAE.

### Ethical approval

Experimental research and field studies on plants were approved by Review Board of Faculty of Agriculture and Natural Resources, University of Mohaghegh Ardabili, Ardabil, Iran. All methods were carried out in accordance with relevant guidelines and regulations. Harvesting the seeds of rangeland plants from the rangeland of Iran is free, however, the permission to harvest the seeds of the *Festuca ovina* from the Sabaland rangelands was obtained from the Department of Natural Resources and Watershed Management of Ardabil province, Iran.

## Results and discussion

### Input variables selection

To create an applicable model, two powerful methods were used: principal component analysis (PCA) and stepwise regression (SWR) (applied a model with a small number of input parameters to account for a large proportion of BY variance as an output). Based on SWR, the six attributes TSW, RWC, CC, LAI, NI, and VA were incorporated in the model (Table 3). The first two PCA components were chosen because their eigenvalues were > ≈ 1. Together, these two variables contributed for more than 86 percent of the variation (Table 4). According to PCA, the eigenvectors for the two initial components for the above six qualities were the largest (justifying over 86% of BY variation) (Table 4). Based on the same results achieved from both IVS techniques (SWR and PCA), these attributes (TSW, RWC, CC, LAI, NI, and VA) were chosen to be the most proper input parameters for both the ANN and MLR models (Table 5).

**Table 5:** The performance model of linear and nonlinear regression to predict the biological yield of *F. ovina.*

Method	R^2a^	RMSE^b^	MAE^c^
Multiple linear regression	0.816	0.107	0.086
Logistic	0.803	0.109	0.609
Gompertz	0.804	0.108	0.157
Exponential	0.751	0.123	0.103
Chapman-Richard	0.810	0.262	0.225
Asymptotic exponential	0.800	0.109	0.088
Quadratic	0.795	0.111	0.089

^a^: determination coefficient; ^b^Root mean square error; ^c^mean absolute error.

### Multiple linear regression (MLR) model development

Although the convenience of linear regression models is the fundamental reason for their usage in research, these models can also accurately predict the output variable, particularly when there is a strong linear relationship between the input and output variables^37,38^. For this aim, six factors chosen among SWR and PCR (i.e., TSW, RWC, CC, LAI, NI, and VA), were included the MLR model (as independent parameters) to forecast the BY (as the dependent parameter) (Table 6). The following formulas were used to forecast BY for all, training, testing, and cross-validation data sets, respectively:

7 $${\text{All\;data:}}\quad {\text{y}} = - 0.{167} + 0.{9}0{\text{9x}}_{{1}} - 0.{\text{536x}}_{{2}} + 0.{4}0{\text{3x}}_{{3}} + 0.{\text{365x}}_{{4}} + 0.{\text{264x}}_{{5}} + 0.{\text{119x}}_{{6}}$$

8 $${\text{Training\;data:}}\quad {\text{y}} = - 0.{143} + {1}.0{\text{4x}}_{{1}} - 0.{\text{581x}}_{{2}} + 0.{\text{421x}}_{{3}} + 0.{\text{384x}}_{{4}} + 0.{\text{252x}}_{{5}} + 0.0{\text{95x}}_{{6}}$$

9 $${\text{Testing}}\;{\text{data:}}\quad {\text{y}} = - 0.{182} + 0.{\text{856x}}_{{1}} - 0.{\text{495x}}_{{2}} + 0.{\text{398x}}_{{3}} + 0.{\text{324x}}_{{4}} + 0.{\text{243x}}_{{5}} + 0.{\text{131x}}_{{6}}$$

10 $${\text{Cross}}\;{\text{validation}}\;{\text{data:}}{\kern 1pt} \quad {\text{y}} = - 0.{144} + 0.{\text{928x}}_{{1}} - 0.{\text{552x}}_{{2}} + 0.{\text{414x}}_{{3}} + 0.{37}0{\text{x}}_{{4}} + 0.{\text{259x}}_{{5}} + 0.{\text{114x}}_{{6}}$$

where y is the biological yield, ${x}_{1}$ is the thousand seed weight, ${x}_{2}$ is the relative water content of leaf, ${x}_{3}$ is the canopy cover, ${x}_{4}$ is the leaf area index, ${x}_{5}$ is the number of florescence, and ${x}_{6}$ is the viability.

**Table 6:** Summary of T-test analysis for regression parameters in different datasets.

Dataset	Variable	df	Parameter estimate	Standard error	t value	Pr >|t|	Tolerance	Variance inflation	R^2^
All	Intercept	1	− 0.167	0.064	− 5.23	< .001	–	–	0.810
	TSW	1	0.909	0.249	3.54	< .01	0.314	1.485	
	RWC	1	− 0.536	0.212	− 7.15	< .01	0.276	2.311	
	CC	1	0.403	0.291	3.01	< .001	0.543	3.771	
	LAI	1	0.365	0.247	6.54	< .001	0.373	4.216	
	NI	1	0.264	0.786	9.21	< .001	0.781	3.128	
	VA	1	0.119	0.798	3.47	< .001	0.421	1.412	
Training	Intercept	1	− 0.143	0.072	− 7.21	< .001	–	–	0.821
	TSW	1	1.04	0.217	1.42	< .001	0.514	3.343	
	RWC	1	− 0.581	0.221	− 3.59	< .001	0.376	3.561	
	CC	1	0.421	0.265	4.35	< .012	0.594	4.751	
	LAI	1	0.384	0.221	5.62	< .01	0.483	3.251	
	NI	1	0.252	0.794	7.18	< .01	0.621	2.415	
	VA	1	0.095	0.790	5.67	< .001	0.451	2.713	
Testing	Intercept	1	− 0.182	0.052	− 8.21	< .01	–	–	0.805
	TSW	1	0.856	0.224	7.41	< .001	0.414	4.021	
	RWC	1	− 0.495	0.207	− 7.15	< .024	0.341	2.179	
	CC	1	0.398	0.285	5.42	< .01	0.621	2.781	
	LAI	1	0.324	0.261	4.25	< .035	0.513	3.51	
	NI	1	0.243	0.762	6.38	< .01	0.639	3.076	
	VA	1	0.131	0.751	2.87	< .01	0.351	1.051	
Cross validation	Intercept	1	− 0.144	0.062	− 7.35	< .001	–	–	0.791
	TSW	1	0.928	0.271	5.24	< .041	0.371	2.307	
	RWC	1	-0.552	0.219	− 5.23	< .01	0.415	3.217	
	CC	1	0.414	0.301	3.28	< .001	0.427	3.251	
	LAI	1	0.370	0.243	9.45	< .01	0.398	4.515	
	NI	1	0.259	0.771	8.54	< .031	0.725	2.914	
	VA	1	0.114	0.786	4.78	< .01	0.443	3.254	

According to above MLR equations (Eq. 7–10), the forecasted value of BY is a linear composition of TSW, RWC, CC, LAI, NI, and VA parameters, such that the sum of the squared deviations of the real and anticipated BY is minimum. These models show how BY changes with TSW, RWC, CC, LAI, NI, and VA, as well as what values of the model's parameters are required to acquire the desired BY value. However, the ability of these models to forecast the BY is contingent on the existence of a strong linear connection among the parameters. An overview of the statistical variables for regression models created using various types of data is shown in Table 6. As can be shown in Table 6, the MLR models were unable to accurately anticipate the BY. Linear regression models are unable to predict performance due to a small number of input parameters or the existence of complex/nonlinear interactions among components^15^. Despite the fact that there is no MLR modeling research with like attributes to estimate BY in this study, it is obvious that the MLR model (with R^2^ = 0.810) cannot accurately forecast BY.

### Artificial neural network (ANN) model development

In order to create an appropriate ANN model with same input and output parameters which were used for the MLR models, ANN models with some of the most essential ANN architectural features were trained and optimized. As shown in Tables 7 and 8, the least amounts of RMSE and MAE and the highest R^2^ values were acquired by the ANN model with 6–7–3–1 structure, the Levenberg–Marquardt as learning algorithm, tansig as transfer function in hidden layers, and pureline transfer function in output layer. Although, Levenberg–Marquardt algorithm requires more memory compared other algorithms, it is a fast algorithm with high accuracy and efficiency, especially for small data samples (i.e. about 100)^39^. Various modeling studies have also concluded that Levenberg–Marquardt is the superior learning algorithm when compared to other algorithms like as Momentum and Conjugate gradient^5,15,32,40^. Tansig's superiority as a non-linear transfer function in our ANN models is most likely due to its characteristics in converting the analyzed input and afterwards conveying it to the output layer. It converts negative to positive infinity input values to a 0 to 1 output range^41^. On the other hand, linear transfer functions like "purelin" perform a basic linear conversion on the analyzed input before passing it to the output layer. However, the type of relationship between the input and output variables is critical in selecting the transfer function, and the higher implementation of nonlinear functions in the present research could be attributed to the nonlinear relationship between BY and input parameters. Nonlinear transfer functions, which cover non-linear fluctuations, have been used more than other transfer functions relying on the feature studied, particularly when there were non-linear correlations across qualities^14,40,42–44^.

**Table 7:** The performance of the artificial neural network model with different training algorithm and transfer to predict biological yield of *F. ovina.*

Training algorithm	Transfer function in hidden layer	R^2a^	RMSE^b^	MAE^c^
Levenberg–Marquardt	tansig	0.960	0.087	0.065
Levenberg–Marquardt	logsig	0.834	0.104	0.095
Levenberg–Marquardt	purelin	0.421	0.145	0.207
Momentum	tansig	0.804	0.215	0.184
Momentum	logsig	0.652	0.277	0.252
Momentum	purelin	0.342	0.452	0.385
Conjugate gradient	tansig	0.623	0.345	0.312
Conjugate gradient	logsig	0.424	0.402	0.384
Conjugate gradient	purelin	0.234	0.542	0.512

^a^determination coefficient; ^b^Root mean square error; ^c^mean absolute error.

**Table 8:** The ten-best topology of applied ANN model to predict biological yield of *F. ovina.*

Topology	R^2a^				RMSE^b^			
	Train	Test	Cross V	All	Train	Test	Cross V	All
6–5–1	0.951	0.953	0.936	0.928	0.085	0.102	0.082	0.092
**6–7-3–1**	**0.962**	**0.958**	**0.953**	**0.960**	**0.077**	**0.083**	**0.090**	**0.087**
6–4–4–1	0.966	0.946	0.948	0.934	0.077	0.089	0.121	0.086
6–5–5–1	0.941	0.931	0.969	0.938	0.092	0.095	0.095	0.112
6–7–1	0.935	0.953	0.930	0.939	0.102	0.121	0.11	0.093
6–7–5–1	0.933	0.946	0.993	0.931	0.089	0.105	0.131	0.109
6–7–6–1	0.938	0.938	0.931	0.936	0.083	0.115	0.097	0.097
6–8–2–1	0.946	0.932	0.911	0.928	0.075	0.096	0.114	0.132
6–8–3–1	0.946	0.934	0.905	0.923	0.091	0.104	0.124	0.088
6–9–3–1	0.932	0.942	0.956	0.933	0.089	0.142	0.134	0.095
6–27–1	0.940	0.924	0.938	0.932	0.076	0.122	0.124	0.111

^a^determination coefficient; ^b^Root mean square error; ^c^mean absolute error.

Significant values are in bold.

Tables 7 and 8 demonstrate that the number of hidden layers and neurons within every layer, as well as the training algorithm and transfer function, all contributed significantly to the total variance in ANN efficiency. However, the complexity of the model depends on the nature of the subject, and an enhance in the number of hidden layers or the number of neurons in each layer does not always indicate an improvement in model efficiency^5^. In general, the ANN model we discovered in this research has a clean topology, which researchers prefer (with one or two hidden layers and a small number of neurons)^5,12,40,45,46^. The presence of significant nonlinear interactions between variables, as well as the creation of a model with an appropriate topology, can lead to such a high-performance forecast^14,32,40^. Mokarram and Bijanzadeh^18^ applied the MLR model in conjunction with two ANN models, the MLP and radial basis function (RBF) models, to estimate biological yield (BY) and stated that the MLP model had the highest R^2^ values for forecast of BY of *Hordeum vulgare*.

According to prior research, ANN modeling approaches are preferred over MLR modelling techniques^15,40,47,48^. This preference appears to be due to ANN modeling techniques' superior ability to capture the extremely nonlinear and complicated relationship between oil content and important parameters^11,15,49^. In predicting soybean yield, Kaul et al.^50^ stated that ANN produces better findings than traditional statistical procedures.

### Comparing MLR and ANN models for forecasting BY

**Figure 5:**
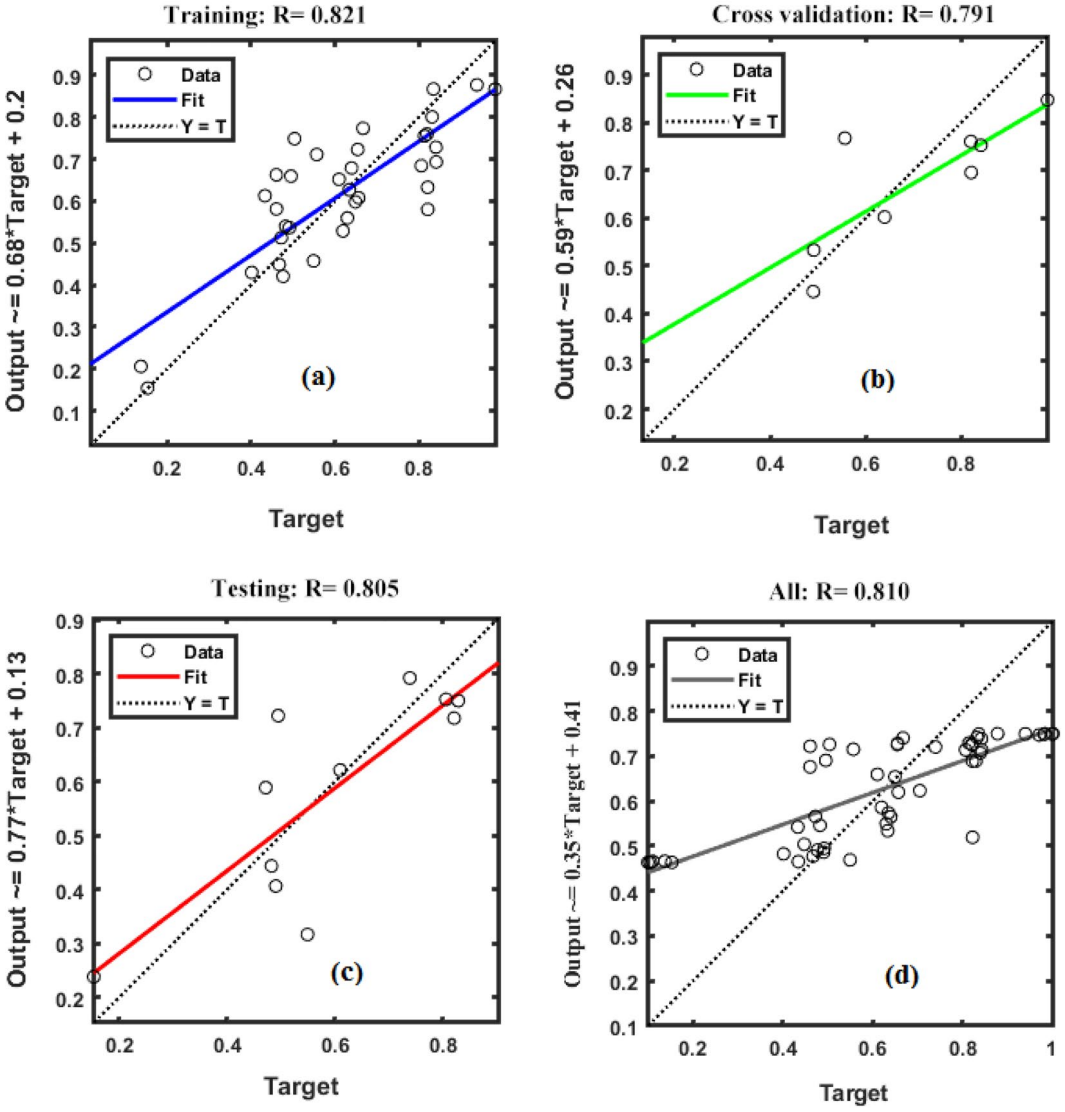
The scatter plot of measured and predicted values of biological yield in fitted multiple linear regression (MLR) model. (**a**) The scatter plot of measured versus predicted values of biological yield in training stage of MLR. (**b**) The scatter plot of measured versus predicted values of biological yield in testing stage of MLR. (**c**) The scatter plot of measured versus predicted values of callus induction percentage in cross-validation stage of MLR. (**d**) The scatter plot of all measured versus all predicted values of biological yield by MLR model.

**Figure 6:**
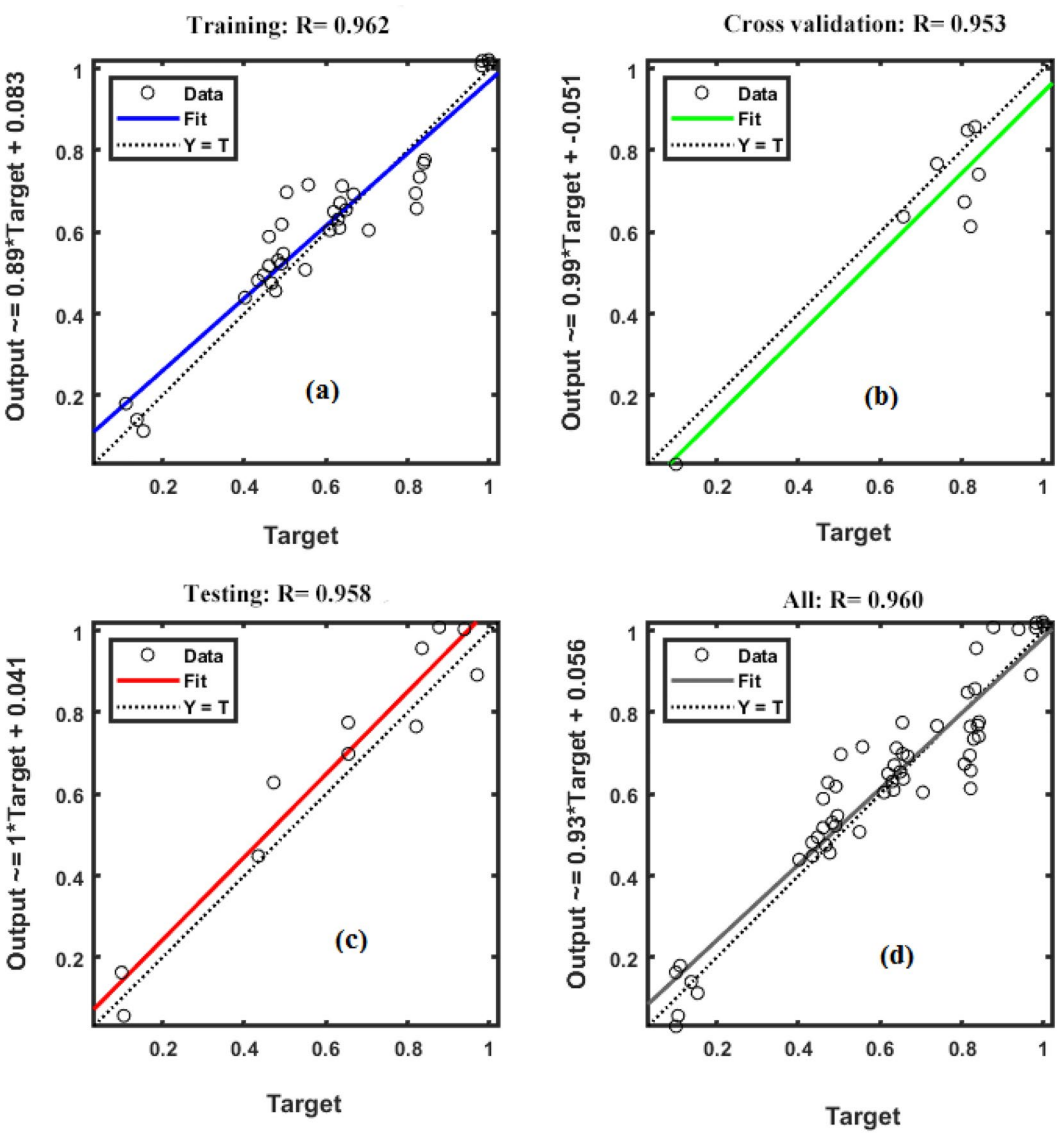
The scatter plot of measured and predicted values of biological yield in fitted artificial neural network (ANN) model. (**a**) The scatter plot of measured versus predicted values biological yield in training stage of ANN. (**b**) The scatter plot of measured versus predicted values of biological yield in testing stage of ANN. (**c**) The scatter plot of measured versus predicted values of biological yield in cross-validation stage of ANN. (**d**) The scatter plot of all measured versus all predicted values of biological yield by ANN model.

To give a more complete contrast between the two modelling techniques for forecasting BY, the models were tested using statistical qualitative metrics and also visual display on scatter plots based on numerous datasets (Figs. 5 and 6). As illustrated in Fig. 5a–d the elected ANN models had higher efficacy to forecast BY than the MLR models (Fig. 6a–d), and compared with MLR models could forecast BY for all, training, testing and cross validation dataset with a 18.52, 17.17, 19 and 20.48% enhance in R^2^ and a decrease of 30.49, 42.86, 37.35, and 32.22% in RMSE, respectively. The use of scatter plots to compare estimated and actual BY values for two models allows for a better understanding of the data distribution and the capability of the selection models to forecast the BY. The observed and forecasted values for the ANN model had the same distribution (with R^2^ = 0.962 and 0.958 for the training and testing datasets, respectively), and the measured values of BY through the ANN model tend to track the matching actual ones very closely (as shown in Fig. 5).

**Figure 7:**
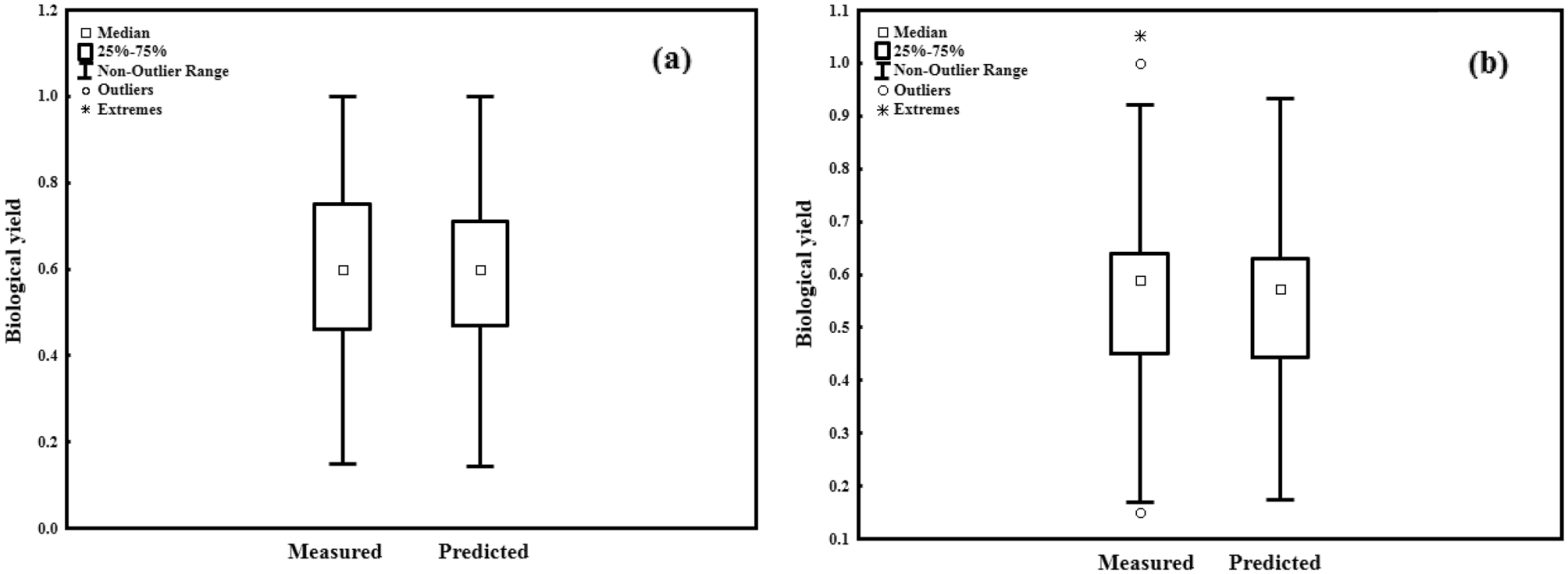
Box plot of measured and predicted BY in the training and testing stages of ANN (**a**, **b**).

**Figure 8:**
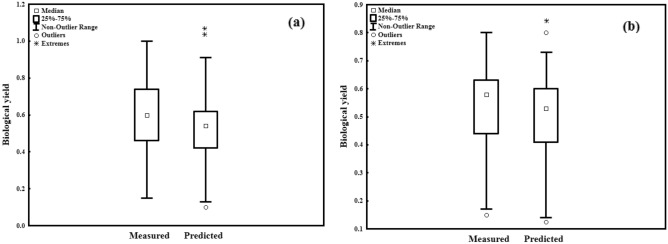
Box plot of measured and predicted BY in the training and testing stages of MLR (**a**, **b**).

**Figure 9:**
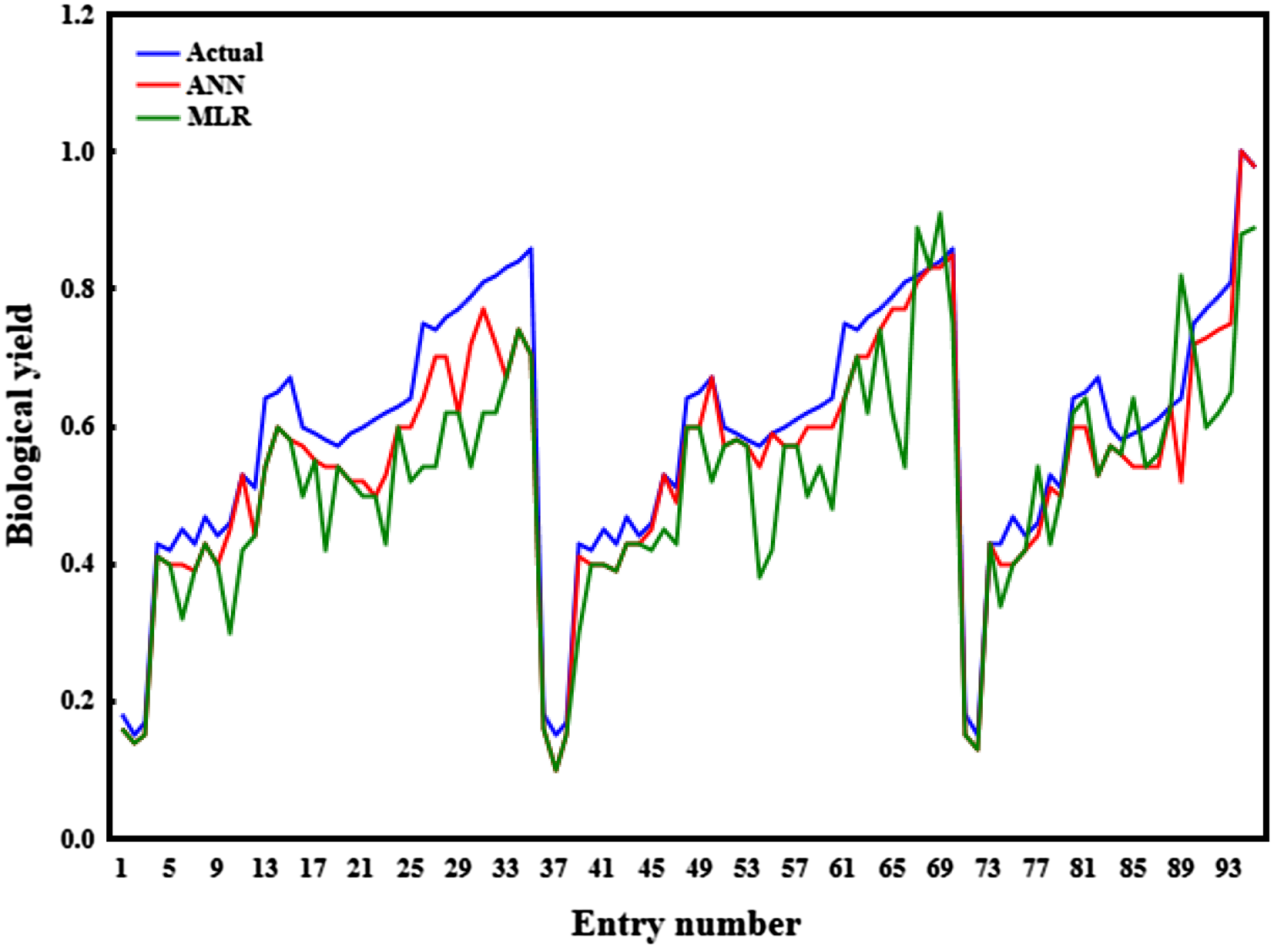
Graphical representation of the measured and predicted values for BY in the training stage of ANN and MLR models.

On the other hand, the estimated and forecasted values for the MLR models, did not have a similar pattern as the ANN models, as demonstrated by the more dispersed distributions and outlines in Figs. 7 and 8. Also, there was no significant difference in statistical summaries (i.e. minimum of sample, lower quartile, median, upper quartile, and maximum of sample) between the observed and measured data by the ANN model, based on box plots (Fig. 7a and b). These characteristics, along with the absence of outliers (unusual data values) in box plots, are necessary modeling characteristics^15,32^. The presence of outlines and variations in the statistical summaries in the box plots generated by the MLR models demonstrated their poor efficacy for predicting BY, in contrast to the ANN models (Fig. 8a and b). It appears that displaying three datasets (the real dataset, data estimated by the ANN, and MLR for BY) on a graph is a better technique for evaluating the efficacy of the two models in forecasting BY. The predicted BY values by the ANN model exhibited a more similar trend to the BY actual values and were more accurate in forecasting BY than the MLR model, as shown in Fig. 9. In general, according to the findings, it can be concluded that ANN models with the same input parameters can forecast the BY (R^2^≈ > 0.95) more accurately than MLR models (R^2^≈ < 0.82). This benefit could be attributed to nonlinear or complicated interactions among variables, as well as nonlinear functions' improved ability to find and take them in ANN models. Contrasting two models in forecasting BY, on the other hand, demonstrated the importance of selecting a model that is appropriate for the subject matter being examined. Many forecasting researches have demonstrated the ANN's benefit in modeling due to its excellent capacity to take highly nonlinear and complex relationships among variables^11,23,40,47,49^.

### Sensitivity analysis

In both ANN and MLR models, the sensitivity tests without a particular input parameter (i.e., TSW, RWC, CC, LAI, NI, and VA) were used to better understand the individual impacts of every input parameter and identify the most and slightest substantial inputs to anticipate BY, and the individual effects of input variables to forecast BY were arranged in Table 9 from highest to lowest. As illustrated in Table 9, both ANN and MLR models without STW had the lowest R^2^ (0.654, 0.504) and the highest RMSE (0.123, 0.142), and MAE (0.102, 0.119), respectively. When the ANN and MLR models are performed without TSW, their ability to predict BY appears to be severely reduced. Based on the sensitivity tests, TSW, RWC, and CC, in that order, were the most crucial variables for forecasting BY in both models. The sensitivity analysis provided a valuable insight of how different variables affected the yield. According to previous studies, TSW are still the most crucial factors having considerable impacts on BY^51^.

**Table 9:** Sensitivity analysis of inputs in the best ANN and MLR models to predict biological yield of *F. ovina* (testing dataset).

Method	ANN			MLR		
	R^2a^	RMSE^b^	MAE^c^	R^2a^	RMSE^b^	MAE^c^
The best ANN/MLR (with TSW, RWC, CC, LAI, NI, VA as input)	0.958	0.083	0.065	0.805	1.114	1.023
MLR/ANN without TSW	0.654	0.123	0.0.102	0.504	1.542	1.241
MLR/ANN without RWC	0.764	0.114	0.095	0.591	1.412	1.213
MLR/ANN without CC	0.783	0.102	0.087	0.673	1.354	1.184
MLR/ANN without LAI	0.821	0.095	0.080	0.710	1.294	1.157
MLR/ANN without NI	0.870	0.090	0.074	0.731	1.263	1.132
MLR/ANN without VA	0.901	0.087	0.069	0.743	1.231	1.107

^a^determination coefficient; ^b^Root mean square error; ^c^mean absolute error.

In addition to the three most important features to forecast BY, other qualities in the model (i.e. LAI, NI, and VA) explained roughly 28.2 percent and 23.1 percent of R^2^ in the ANN and MLR models, respectively. Based on the findings of the sensitivity testing, cultivars having early blooming, prolonged maturity, and a high TSW are excellent for enhancing BY when designing the proper structure. These early blooming cultivars are not likely to experience stress at this important phase, and if they are not exposed to drought and warm tension at the end of the growing season under rainfed conditions, they can continue their vegetative and reproductive growth.

## Conclusion

In harsh environmental conditions, such as rainfed areas under heat and drought stress, the inheritance of quantitative polygenic features such BY is decreased significantly. In such circumstances, the genetic benefit of selection is decreased, and direct selection has a poor effect on the targeted feature. Applying modeling techniques to determine and combine indirect selection indications will help create a favorable design for the targeted feature, which is one way to overcome this difficulty. In order to achieve this, we created an ANN model as well as an MLR model to forecast BY using attributes chosen using PCA and SWR techniques (i.e. TSW, RWC, CC, LAI, NI, and VA). According to the findings, the ANN model with the Levenberg–Marquardt learning algorithm, tansig transfer function, and two hidden layers (i.e. structure 6–3–7–1) predicted the BY more accurately than the MLR. The capacity of the ANN model to detect complex and nonlinear effects, as opposed to the MLR model, may explain the advantage of the ANN model in forecasting BY. The ANN model could be a favorable alternative to classic modeling approaches like as path analysis, regression, and so on, due to the significant difference in efficacy of the two models in forecasting BY. Sensitivity analysis for both models revealed that STW was the most influential component in predicting BY, followed by RWC and CC, respectively. So, genotypes of sheep fescue with early flowering, long maturation, and high TSW are optimal for increasing BY. It appears that designing breeding strategies to produce plants with the above structure could open up a new window in the future evolution of this plant.

## Data Availability

The datasets generated during and/or analysed during the current study are available from the corresponding author on reasonable request.
