# Author Correction: An efficient estimation of crop performance in sheep fescue (*Festuca ovina* L.) using artificial neural network and regression models

**DOI:** 10.1038/s41598-023-30816-4

**Published:** 2023-03-03

**Authors:** Masoomeh Abbasi Khalaki, Esfandiar Jahantab, Moslem Abdipour, Mehdi Moameri, Ardavan Ghorbani

**Affiliations:** 1 Department of Range and Watershed Management, University of Mohaghegh Ardabili, Ardabil, Iran; 2 Department of Range and Watershed Management, Faculty of Agriculture, Fasa University, Fasa, Iran; 3 Kohgiluyeh and Boyerahmad Agricultural and Natural Resources Research and Education Center, Agricultural Research, Education and Extension Organization (AREEO), Yasuj, Iran; 4 Department of Plant and Medicinal Plants, University of Mohaghegh Ardabili, Ardabil, Iran

Correction to: *Scientific Reports* 10.1038/s41598-022-25110-8, published online 28 November 2022

In the original version of this Article, Moslem Abdipour was omitted as a corresponding author. Correspondence and requests for materials should also be addressed to abdipur.m@gmail.com.

The original Article has been corrected.

